# Oxygen Therapy in Headache Disorders: A Systematic Review

**DOI:** 10.3390/brainsci11030379

**Published:** 2021-03-17

**Authors:** Tiziana Ciarambino, Gennaro Sansone, Giovanni Menna, Ombretta Para, Giuseppe Signoriello, Laura Leoncini, Mauro Giordano

**Affiliations:** 1 Internal Emergency Department, Hospital of Marcianise, 80125 ASL Caserta, Italy; tiziana.ciarambino@gmail.com; 2 Department of Advanced Medical and Surgical Sciences, University of Campania, L. Vanvitelli, 80138 Naples, Italy; gennarosansone90@gmail.com (G.S.); mennagiovanni@hotmail.it (G.M.); Giuseppe.signoriello@unicampania.it (G.S.); 3 Internal Emergency Department, Careggi Hospital University of Florence, 50012 Florence, Italy; ombretta.para@gmail.com; 4 Hospital of Marcianise, 80125 ASL Caserta, Italy; Lauraleoncini@inwind.it

**Keywords:** oxygen therapy, headache, migraine

## Abstract

Background: The global active prevalence of migraines is approximately 14.7%. Oxygen therapy may reduce the use of non-steroidal anti-inflammatory drugs (NSAIDs) which often have various negative side effects. The purpose of this systematic review is to analyze the literature on the efficacy of high flow oxygen for the management of headache disorders, compared to placebo treatment. Methods: Studies were identified by PubMed, Web of Science and Scopus database from 1980 to the 30 October 2020. The search included the following terms: “oxygen therapy” and “headache” and “migraine”. Studies were included if high flow oxygen was used in the treatment of headache disorders. All selected studies were qualitatively analyzed. Results: Our literature search identified 71 studies, of which 65 were discarded and 6 were included in the meta-analysis. The random effect model did not show a pooled significant resolution of headache disorders (OR 2.08 (95% CI 0.92–4.70), *p* < 0.0001) in the oxygen therapy group compared to the placebo group. Conclusion: In our systematic review of six studies, there were no significant differences between high flow oxygen and placebo treatment groups.

## 1. Introduction

Primary headache disorders, which include migraines, cluster headaches and tension type headaches, are a common disorder, which can lead to significant disability world-wide, with prevalence between 0.1 and 20% [1,2]. Headache disorders have always been treated with various oral medications. In particular, we will focus, in this systematic review, on a particular acute treatment, high flow oxygen. This therapeutic approach has been studied for several decades. In fact, the first trial to show efficacy of oxygen inhalation via a mask was conducted in 1940 [3]. As described in the literature, there are many scientific reasons why oxygen might be effective in treating acute migraines. In this regard, abnormal oxygen utilization, tissue hypoxia, and cerebrovascular dysfunction are implicated in the pathogenesis of migraine headaches [4,5]. Experimental hypoxia induces migraines with and without aura [6,7] whereas hyperoxia has vasoconstrictive effects [8]. Although oxygen therapy may not directly suppress cortical depression [9], it may suppress depression triggered by micro-embolism [10], which is believed to be a migraine trigger [11]. Indeed, oxygen therapy inhibits peri-infarct spreading depolarizations [12], reduces inflammation and blood–brain barrier damage in animal models of migraine [13], and may have yet other mechanisms similar to those documented in models of ischemic stroke [5]. Different studies reported in the literature demonstrated that the effectiveness of oxygen was comparable to other therapy (triptans for example) and under-line that oxygen therapy has a better tolerability profile [14,15]. The purpose of this systematic review is to analyze the literature on the efficacy of high flow oxygen, compared to placebo treatment of migraines.

## 2. Methods

**Figure 1:**
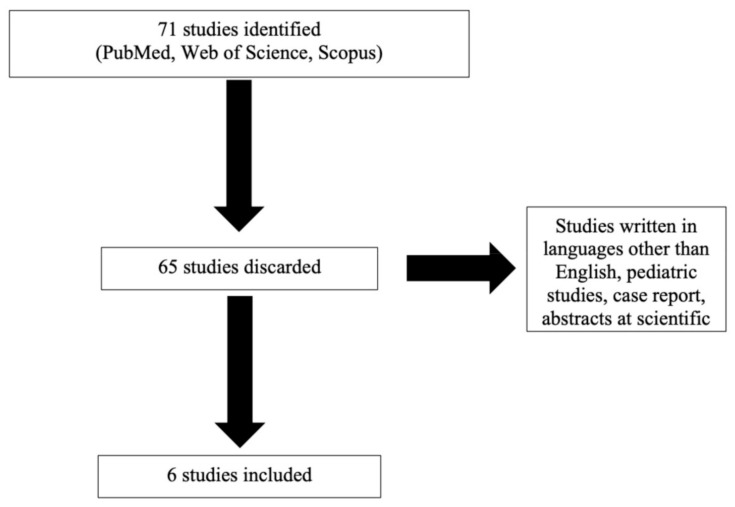
Research Studies.

Studies were identified by PubMed, Web of Science and Scopus database from 1980 to 30 October 2020. The search included the following terms: “oxygen therapy” and “headache” and “migraine.” Bibliographies of recent review articles and references of articles included were manually searched to identify additional studies. After the studies were selected, their references were reviewed for potential inclusion. Studies written in languages other than English, pediatric studies, case report, abstracts at scientific conferences were excluded (Figure 1). Three authors (T.C., G. Sansone and G.M.) reviewed all study abstracts.

### 2.1. Study Selection

Randomized and observational studies were included if they:

1. Enrolled adult patients (≥18 years old);
2. Included patients on oxygen therapy vs. placebo therapy;
3. Integrated the adjusted Odds ratio (aOR) of remission in the oxygen therapy group compared to the placebo therapy group or could be calculated from the paper.

### 2.2. Data Extraction and Quality Assessment

Data extracted from the identified studies included clinical setting, headache definition, inclusion and exclusion criteria, number of participants, age, ward of admission, and concomitant drugs. Titles and abstracts of full-texts obtained from the researches were analyzed by three authors independently (T.C, G.M. and G. Sansone). The Newcastle–Ottawa Scale (NOS) was used for assessing the quality of each study [16]. The NOS assigns up to a maximum of nine points in three domains:

1. selection of study groups (four points);
2. comparability of groups (two points);
3. ascertainment of exposure and outcomes (three points) for case–control and cohort studies, respectively. Dissents in the evaluation of the quality were resolved by consensus. Studies with ≥6 points were included in this review.

### 2.3. Outcomes

The main outcomes were the headache resolution in adult patients who received the oxygen therapy compared to those who received placebo treatment. This outcome was evaluated for all studies for which an adjusted Odds Ratio (aOR) was calculated. Since the definition of headache could differ across the studies, the definition of headache used in the primary outcome analysis from each reviewed study was used. No secondary outcomes were examined. Headache disorders were defined based on literature research [2].

### 2.4. Summary Measures

Each study contained results of a multivariable logistic regression model with the estimation of adjusted Odds Ratio.

### 2.5. Synthesis of Results

Statistical analysis was performed using R foundation for statistical computing (version 3.6.0; 26 April 2019) with the “meta-phor” R package version 2.1.0 (13 May 2019). Statistical significance alpha was fixed to 0.05. Considering the methodological difference of included studies, a random effects model using the generic inverse variance method for pooling results was preferred. The model was fitted using the Restricted Maximum Likelihood (REML) method, with the estimation (95% CI) of summary OR, Q for heterogeneity, and I^2^ (total heterogeneity/total variability).

## 3. Results

### 3.1. Study Selection and Characteristics

Our literature search identified 71 studies, of which 65 were discarded and 6 were included in the meta-analysis. We excluded 65 records because they did not include relevant reports or data. Six articles were eligible per inclusion criteria [5,14,15,17,18,19] as reported in Table 1. For the studies included in the meta-analysis, an aOR was provided. These include three randomized controlled trials (RCTs), one cohort study and two survey studies. The six studies were of good methodological and statistical quality with low risk of bias. All of the individual studies did show a significant resolution of headache disorders (Test of OR = 1, z = 1.76, *p* = 0.078) for the oxygen therapy group compared to the placebo group.

### 3.2. Synthesis of Results

**Figure 2:**
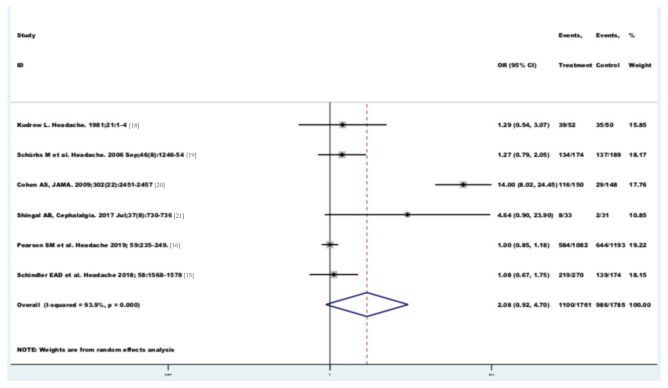
Forest plot of the pooled odds ratio (OR) of migraine in the oxygen therapy group compared to the placebo group, (*p* = 0.000). I^2^ = I-squared (variation in OR attributable to heterogeneity) = 93.9%.

The random effect model (Figure 2) did not show a pooled significant resolution of headache disorders (OR 2.08 (95% CI 0.92–4.70), *p* < 0.0001) in the oxygen therapy group compared to placebo group. Heterogeneity chi-squared was 82.30 (df = 5) *p* = 0.000. I-squared (variation in OR attributable to heterogeneity) = 93.9%. The estimate of between-study variance Tau-squared was 0.8899.

### 3.3. Results

In Table 1 we summarize the studies included in this review. Kudrow L (1981) showed how the use of oxygen therapy for cluster headaches obtained excellent results [17]. Schürks M et al. (2006) reported that oxygen was the most effective acute treatment for cluster headaches [18]. In 2009, in another study, 26% of patients treated with high flow oxygen were pain free at 15 min and 24% were pain free at 30 min, compared to 7% and 8%, respectively, for those treated with a placebo treatment [19]. In 2016, the Singhal study reported a non-significant difference between the oxygen-treated group and the air-treated group. In particular, the mean decrease in pain score from baseline to 30 min, was 1.38 +/− 1.42 in the oxygen-treated group and 1.22 +/− 1.61 in air-treated group. The authors reported a complete resolution of migraine in 24% of the attacks treated with oxygen versus 6% in those treated with a placebo [5]. Schindler et al. (2018) and Pearson SM et al. (2019), conducted two surveys on the use of oxygen for patients with cluster headaches. They demonstrated that the effectiveness of oxygen was comparable to triptans and emphasized that oxygen therapy had a better tolerability profile [14,15].

## 4. Discussion

High flow oxygen therapy for migraine is a reality, potentially with a better tolerability profile than other therapies. It has been reported that oxygen therapy is a safe and low-cost therapy [20]. It is interesting that high flow oxygen therapy used in the treatment of headaches in Emergency Departments may reduce the use of non-steroidal anti-inflammatory drugs (NSAIDs) that can expose patients to various side effects. Oxygen can have quite rapid effects as reported by the Ozkurt and Cohen study [19,20,21]. Another crucial issue, that can be deduced from the analysis of these studies, is the latency of effect. In particular, at 15 min post treatment, there was a reduction or resolution of symptoms in the high flow oxygen group [19,21].

The findings from this systematic review and meta-analysis, of an adult population with migraine, suggest that oxygen therapy is not associated with significantly different outcomes compared to placebo therapy. There was heterogeneity between studies as evidenced by the I^2^ value of 93.9%. Although a random effects model was used to account for the heterogeneity, related to different baseline characteristics of patients, variables of OR adjustments between included studies, and the presence of concomitant drugs, the duration and concomitant use of specific drugs could have affected the remission of headache in these studies.

Additional RCT studies are needed to evaluate the reliability and effectiveness of high flow oxygen therapy in the management of migraine before high flow oxygen therapy can be included in guidelines for the management of migraine in the emergency department.

### 4.1. Key Messages

High flows oxygen treatment of headaches

- Is a reality right now, especially for the treatment of cluster headaches;
- May have a better tolerability profile and be a safe and low-cost therapy;
- Can have rapid effects (after 15 min);
- May reduce the use of NSAIDs;
- May be useful to solve acute migraine and associated symptoms.

### 4.2. Limitations

Only six studies were included in this systematic review. Few studies described outcomes objectively (e.g., Visual Analogue Scale (VAS)) score or the reduction in the symptoms related to the headache). As described in Table 1, few studies reported the number of patients with headache attacks treated or not treated, the number of patients treated with high flow oxygen or placebo treatment or the number of patients in both groups (oxygen or placebo) in relation to outcome.

### 4.3. Conclusions

This systematic review of the treatment of headache disorders suggests there is no significant difference in efficacy of high flow oxygen or placebo treatment for the management of headache disorders.

## Figures and Tables

**Table 1:** Included studies.

Authors, Journal	Study Design	n. Pts	Age	n. Male	n. Attacks Treated	n. Oxygen Treated	n. Placebo Treated	n. Oxygen/Tot	n. Placebo/Tot	Outcome Oxygen	Outcome Placebo	Type of Oxygen	NOS
Kudrow L, Headache 1981 [17]	RCT	102	48.5	45	NA	52	50	NA	NA	75% successfully treated CH attacks	70% successfully treated with ergotamine	100%	6
Schürks M, Headache 2006 [18]	Cohort Study	246	44.8	77.6%	NA	71.1%	77.6% of pt used triptans	NA	NA	76.6% effectiveness of oxygen	71.7% effectiveness of triptans	NA	6
Cohen AS, JAMA 2009 [19]	RCT	76	39 +/− 10	64	298	150	148	150/298	148/298	Pain free 15 min: 78% 30 min: 72%	Pain free 15 min: 20% 30 min: 24%	100%	6
Shingal AB, Cephalalgia. 2017 [5]	RCT	22	36 +/− 10	12	64	33	31	33/64	31/64	Pain free: 24%	Pain free 6%	100%	6
Schindler EAD, Headache 2018 [14]	Survey	493	NA	367	NA	270	174 pt used sumatriptan	NA	NA	81.5% of pt had good response with oxygen	80.5% of pt had good response with sumatriptan	Oxygen flow >10 l/min	6
Pearson SM, Headache 2019 [15]	Survey	2193	46	1104	1604 CH	1082	1193 used triptans	NA	NA	54% oxygen was effective or very effective	54% triptans was effective or very effective	NA	6

RCTs: randomized controlled trials. NOS: The Newcastle–Ottawa Scale. NA: not applicable. CH: headache. pt: patients.
